# Supplementary material for: Refining the Intraoperative Identification of Suspected High-Grade Glioma Using a Surgical Fluorescence Biomarker: GALA BIDD Study Report
Source: J Pers Med. 2023 Mar 13;13(3):514. doi: 10.3390/jpm13030514 (PMC10058333; doi:10.3390/jpm13030514)
Supplement: Supplementary file 1 [file jpm-13-00514-s001.zip › jpm-2253883-supplementary.pdf]

SUPPLEMENTARY FIGURES & TABLES

Table S1. Baseline Characteristics of patients analysed in final analysis and excluded.

Characteristics	Analysed (N=89)	Excluded (N=17)
Age, n/N (%)		
>=20 and <30	2/89(2.2)	
>=30 and <40	8/89(9)	3/17(17.6)
>=40 and <50	9/89(10.1)	3/17(17.6)
>=50 and <60	24/89(27)	5/17(29.4)
>=60 and <70	32/89(36)	3/17(17.6)
>=70 and <80	14/89(15.7)	3/17(17.6)
Sex, n/N (%)		
Male	51/89(57.3)	12/17(70.6)
Female	38/89(42.7)	5/17(29.4)
WHO Performance status		
0	40/88(45.5)	14/17(82.4)
1	48/88(54.5)	3/17(17.6)
No. of tumour locations		
1	86/89(96.6)	15/17(88.2)
2	3/89(3.4)	2/17(11.8)
Tumour details		
Front	33/92(35.9)	7/19(36.8)
Temporal	36/92(39.1)	4/19(21.1)
Parietal	16/92(17.4)	4/19(21.1)
Occipital	4/92(4.3)	1/19(5.3)
Multifocal	3/92(3.3)	3/19(15.8)
Tumour Hemisphere		
Left	47/89(52.8)	6/17(35.3)
Right	42/89(47.2)	11/17(64.7)
WHO grade evaluated by local radiologist		
WHO grade II		1/17(5.9)
WHO grade IV	77/89(86.5)	7/17(41.2)
WHO grade II transforming to a higher grade	12/89(13.5)	8/17(47.1)
Other (Not specified)		1/17(5.9)

Table S2. Molecular pathology of patients by visible fluorescence status and diagnosis (WHO 2021)

Variables		WHO grade I	WHO grade II	WHO grade III	WHO grade IV	Total
**Visible fluorescence, N**			**1**	**3**	**77**	**81**
IDH-1 mutation, n/N (%)						
	No			2/3(66.7)	75/76(98.7)	77/80(96.3)
	Yes		1/1(100)	1/3(33.3)	1/76(1.3)	3/80(3.8)
MGMT methylation status, n/N (%)						
	Methylated			2/3(66.7)	29/75(38.7)	31/78(39.7)
	Unmethylated			1/3(33.3)	46/75(61.3)	47/78(60.3)
1p19q co-deletion, n/N (%)						
	No			1/2(50)	9/9(100)	10/12(83.3)
	Yes		1/1(100)	1/2(50)		2/12(16.7)
**Without Visible fluorescence, N**		**1**	**7**			**8**
IDH-1 mutation, n/N (%)						
	No	1/1(100)	1/6(16.7)			2/7(28.6)
	Yes		5/6(83.3)			5/7(71.4)
MGMT methylation status, n/N (%)						
	Methylated		2/3(66.7)			2/3(66.7)
	Unmethylated		1/3(33.3)			1/3(33.3)
1p19q co-deletion, n/N (%)						
	No		2/5(40)			2/5(40)
	Yes		3/5(60)			3/5(60)
